# Targeting the receptor binding domain and heparan sulfate binding for antiviral drug development against SARS-CoV-2 variants

**DOI:** 10.1038/s41598-024-53111-2

**Published:** 2024-02-02

**Authors:** Zi-Sin Yang, Tzong-Shiun Li, Yu-Sung Huang, Cheng-Chung Chang, Ching-Ming Chien

**Affiliations:** 1 Department of Medical Sciences Industry, College of Health Sciences, Chang Jung Christian University, Tainan, 711 Taiwan; 2 Institute of Bioinformatics and Structural Biology, National Tsing Hua University, Hsinchu, 300 Taiwan; 3 Graduate Institute of Biomedical Engineering, National Chung Hsing University, Taichung, 402 Taiwan; 4 Department of Plastic Surgery, Chang Bing Show Chwan Memorial Hospital, Changhua, 500 Taiwan

**Keywords:** Infectious diseases, Drug discovery and development, Computational biology and bioinformatics, Drug discovery

## Abstract

The emergence of SARS-CoV-2 variants diminished the efficacy of current antiviral drugs and vaccines. Hence, identifying highly conserved sequences and potentially druggable pockets for drug development was a promising strategy against SARS-CoV-2 variants. In viral infection, the receptor-binding domain (RBD) proteins are essential in binding to the host receptor. Others, Heparan sulfate (HS), widely distributed on the surface of host cells, is thought to play a central role in the viral infection cycle of SARS-CoV-2. Therefore, it might be a reasonable strategy for antiviral drug design to interfere with the RBD in the HS binding site. In this study, we used computational approaches to analyze multiple sequences of coronaviruses and reveal important information about the binding of HS to RBD in the SARS-CoV-2 spike protein. Our results showed that the potential hot-spots, including R454 and E471, in RBD, exhibited strong interactions in the HS-RBD binding region. Therefore, we screened different compounds in the natural product database towards these hot-spots to find potential antiviral candidates using *LibDock*, *Autodock vina* and furthermore applying the MD simulation in *AMBER20*. The results showed three potential natural compounds, including Acetoside (ACE), Hyperoside (HYP), and Isoquercitrin (ISO), had a strong affinity to the RBD. Our results demonstrate a feasible approach to identify potential antiviral agents by evaluating the binding interaction between viral glycoproteins and host receptors. The present study provided the applications of the structure-based computational approach for designing and developing of new antiviral drugs against SARS-CoV-2 variants.

## Introduction

Severe Acute Respiratory Syndrome Coronavirus 2 (SARS-CoV-2), a type II coronavirus, caused a pandemic of acute respiratory illness starting in late 2019, leading to a severe public health crisis and economic losses worldwide^1^. Notably, the emergence of new SARS-CoV-2 variants due to genetic mutations has raised concerns about the effectiveness of COVID-19 antiviral drugs and vaccines^2,3^. SARS-CoV-2 variants can lead to changes in binding sites that may affect the ability of the virus to bind to specific drug targets, reducing the efficacy of antiviral drugs or rendering them completely ineffective against the mutant strains^4^. As new variants of the virus evolve, it becomes increasingly important to understand the disease and drug targets^5^. For example, the Omicron variant has a remarkably high transmission rate, detected in 155 countries^5^. There is an urgent need to explore antiviral therapeutic targets and effective clinical drugs^6,7^. Therefore, the most severe outbreaks of SARS-CoV-2 highlight the importance of coronaviruses as human pathogens and emphasize the need to develop new antiviral strategies to combat acute respiratory illness caused by SARS-CoV-2 variants.

The particles of SARS-CoV-2 have an irregular shape containing an outer envelope with distinctive, 'club-shaped' peplomers that give the virus a crown-shaped appearance (corona)^8^. The viral genome of SARS-CoV-2 consists of a single-stranded positive-sense RNA of about 30 kb. It contains several genes coding for various structural and non-structural proteins required for the production of virion progeny^9^. The virion of the SARS-CoV-2 envelope surrounding the nucleocapsid contains the following structural proteins: spike (S), membrane (M), envelope (E), and nucleocapsid (N) protein^10^. In particular, the trimeric spike glycoprotein (S protein) of SARS-CoV-2 is a crucial target for virus neutralization^11^. Several therapeutic targets are repurposed against COVID-19 and viral elements used in COVID-19 vaccine candidates^12^. The S protein covering the surface of SARS-CoV-2 binds to the host receptor angiotensin-converting enzyme 2 (ACE2), leading to the entry of the virus into the cell^13^. COVID-19 variants often alter critical viral proteins, particularly the S protein, which is responsible for viral entry into host cells. Many mutations in the emerging variants are located in the receptor-binding domain (RBD) of the S protein, which is targeted by most monoclonal antibodies from COVID-19 patients^14^.

Heparan sulfate (HS) is a linear polysaccharide found on the surface of cells and plays a critical role in several biological processes, including cell signaling, adhesion, and viral attachment^15^. Viruses use HS interactions to enhance attachment to the surface of host cells and improve their chances of interacting with specific entry receptors^16–18^. In COVID-19 infection, HS serves as a co-receptor and facilitates the binding of the viral S protein to the host cell^19^. The S protein of the SARS-CoV-2 virus recognizes and attaches to HS molecules present on the surface of host cells. This interaction triggers a conformational change in the S protein, exposing the RBD that allows the virus to attach to the ACE2 receptor on the host cell membrane. This binding event is essential for the subsequent internalization of the virus into the host cell and initiating the infection process. Furthermore, HS has been shown to be essential for the entry of viruses into host cells through its interaction with ACE2 and the RBD of the S protein of SARS-CoV-2^20^. Hence, the development of compounds that effectively interfere with the binding of HS to the RBD in the viral S protein presents a good strategy against SARS-CoV-2 infection.

In the filed of drug discovery against SARS-CoV-2 infection, many research articles have reported on multiple computational techniques^21–25^. These computational approaches were applied, such as molecular docking, molecular dynamics, and digital genetic sequencing. These methods were incorporated into drug-targeting strategies in bioinformatics, genomics, and proteomics^26–29^. Previously, there has been a predominant focus on targeting particular proteins, such as Mpro^30^, papain-like protease^31^, RBD^32^, or host-ACE2^33^. Recntly, several natural products showed excellent antiviral activities by targeting structural and non-structural proteins of SARS-CoV-2^34–36^. Therefore, the use of natural products can be a good option for drug development.

NRICM101 a traditional Chinese medicine formula developed by the National Research Institute of Chinese Medicine (NRICM) in Taiwan. This formula had successfully shown antiviral activities to prevent SARS-CoV-2 infection^37^. This natural herbal-based formula was proposed by NRICM to target viral respiratory infection and immunomodulation during the SARS CoV outbreak in 2003^38,39^. One of the pharmaceutical functions of NRICM101 was proposed to interfere with host cell invasion and viral replication by binding the viral spike protein^40^. However, little is known about the active compounds in NRICM101 in suppressing SARS-CoV-2.

In this report, we used computational approach to analyze multiple sequences of coronaviruses and reveal important information about the binding of HS to RBD in the SARS-CoV-2 S protein. First, we identified the conserved 'hot-spots' on the RBD as potential targets for the development of new antiviral drugs. A total of 1382 herbal compounds from the natural chemical formulation of NRICM101 were docked to the identified hot-spots as potential antiviral drug candidates using in silico methods. Our results indicate the possible mechanism of NRICM101 regarding the active ingredients in suppressing SARS-CoV-2 and may provide useful information for the design and development of new antiviral agents against SARS-CoV-2 and other pandemic diseases.

## Materials and methods

### Structure and sequence preparation of RBD in SARS-CoV-2 spike protein

The X-ray crystal structures and sequences of RBD in different species different species (Guangxi pangolin, PDB ID: 7DDP; Guangdong pangolin, PDB ID: 7DDO; RaTG13, PDB ID: 7TTX; BANAL-236, PDB ID: 7PKI) and different variants of SARS-CoV-2 S protein (wild type, PDB ID: 7DDD; Beta, PDB ID: 7PRY; Gamma, PDB ID: 7M8K; Delta, PDB ID: 7W92; Kappa, PDB ID: 7VXI; Epsilon, PDB ID: 7N8H; Omicron, PDB ID: 7TB4) were retrieved from the Research Collaboratory for *Structural Bioinformatics (RCSB) protein data bank (PDB)* (https://www.rcsb.org/). Protein preparation was carried out in *PyMOL* (The PyMOL Molecular Graphics System, Version 2.3.2, Schrödinger, LLC.) by removing water molecules, any co-crystallized compounds, and other unnecessary regions. The amino acid region from 336 to 515 was selected for further in silico computational studies^41^. Multiple structure and sequence alignments of the RBDs of all selected multi-species coronavirus S protein and variants of the SARS-CoV-2 S protein were performed using *PyMOL, ConSurf online server* (https://consurf.tau.ac.il/)^42^. The *Weblogo online tool* was used to display the consensus sequences from the multiple sequence alignments of all RBDs of SARS-CoV-2 S protein (https://weblogo.berkeley.edu/logo.cgi).

Identifying the potential binding sites (hot-spots) involved in the interaction is an important step in docking to test new chemical substances. The residues in the active site form a pocket containing a variety of hydrogen acceptors, donors, hydrophilic regions, and hydrophobic regions. To determine the binding cavity in the RBD of the SARS-CoV-2 spike protein, protein preparation was performed in *PyMOL* by removing water molecules, any co-crystallized compounds, and other unnecessary regions. The highly conserved region Y453 to G476 of the RBD of the wild-type SARS-CoV-2 S protein was selected and docked to HS using the LibDock program in *Discovery studio client v20.1.0.19225 (Discovery studio 2019)* to predict the key active site residues in the RBD of the wild type SARS-CoV-2 protein for binding with HS in terms of hydrogen bonding and hydrophobic interaction^37,38^.

### Preparation of natural compounds from NRICM101

Thousands of important compounds from ten medicinal plants in NRICM101 (*Scutellaria baicalensis*, *Houttuynia cordata*, *Morus alba*, *Saposhnikovia divaricata*, *Trichosanthes kirilowii*, *Isatis indigotica*, *Glycyrrhiza glabra*, *Magnolia officinalis*, *Mentha haplocalyx* and *Nepeta tenuifolia*)^39^ were used for a virtual screening and molecular docking study against RBD of SARS-CoV-2 target proteins. The structures of compounds were retrieved from *PubChem*^40^ and *ZINC15*^43^ databases, and verified the protonation state of compounds, then finally merged with *Open Babel* software for molecular docking^43^.

### Molecular docking

The binding pocket with X: 245.6069; Y: 216.0802; Z: 197.8738 in a radius 11.49 Å containing residues R454, F456, R457, S459 and E471 of the RBD of the wild-type SARS-CoV-2 S protein (PDB ID: 7DDD) was selected as the binding site for screening compounds that could potentially inhibit the RBD. Otherwise after molecular docking, the binding energies for compound-receptor interactions were calculated by ‘Binding Energy’ protocol^44^ of *Discovery studio 2019* Virtual screening was performed using the LibDock^45^ and binding energies module of *Discovery studio 2019*. LibDock is a rigid-based docking module. It calculates hot-spots for the protein using a grid placed in the binding site and polar and apolar probes. The hot-spots are then used to align the compounds to form favorable interactions. Virtual screening was performed by docking all prepared compounds to the defined active site using LibDock. Based on the LibDock scores, all docked poses were ranked and grouped, and all compounds were also ranked according to the LibDock scores. Subsequently, the compounds were filtered out if the LibDock score > 80 and met the criteria of Lipinski's rule of 5 (logP: − 2–5). The second platform for molecular docking was applied *Autodock vina* as the second platform for molecular docking. All targets were loaded in the *AutoDockTools-1.5.7* including RBD, Acetoside (ACE), Isoquercitrin (ISO), Hyperoside (HYP), Chrysin 6-C-arabinoside 8-C-glucoside (CAG), Oroxyloside (ORO), and Chrysin 6-C-glucoside 8-C-arabinoside (CGA) to convert into pdbqt format; a prerequisite for *Vina Autodock* software. The binding pocket that X: 245.6344; Y: 217.076; Z: 197.1578 with grid box sized 20 × 20 × 20 Å surrounding the binding site of RBD. Docking procedure was repeated over ten times with exhaustiveness set at 8^46^. Finally, the six best potential compounds with *Libdock* binding energies <  − 200 kcal/mol and more than 3 hydrogen bonds with the active site residues determined by LigPlot+ ^47^ were selected for the molecular dynamics (MD) simulation^48^.

### MD simulation

The best binding complex conformations of RBD with top six compounds (Table S1) and HS were selected for molecular dynamics simulations (MD) to determine and compare the stability of the complex. To generate the initial structure for MD simulation of RBD complexes with top six compounds and HS, we establish the force field for top six compounds and HS by the *antechamber* of *AmberTools20*^49^**.** In addition, these RBD complexes were solvated in a cubic box of TIP3P water model. The size of the simulation system was enough to ensure at least 10 Å between the protein and the edge of the simulation box. The force field of ff14SB^50^, GAFF2, and the water model TIP3P were used for the *tLEaP* of *AmberTools20*^49^. Each MD simulation for RBD complexes were done by *AMBER20* with three independent repeats^49^. The minimization is calculated of 2500 steps of steepest descent and then 2500 steps of conjugate gradient in all 3 sections. The first section constrains all atoms of protein. In the second section, the atoms of backbone were constrained, the water, lipid and the side chains of protein will be minimized. In the third section, the entire system was optimized without any constraint. Then, the simulation systems were heated from 0 to 300 K, at 100 ps and equilibrated by NVT ensemble calculation method, in which the total number of atoms, volume and temperature are fixed, at 100 ps. Finally, the production of each MD simulations of 100 ns were performed from the NPT ensemble calculation method, in which the total number of atoms are fixed and at 300 K and 1 atm. Trajectory analysis was performed using the analysis tools by Cpptraj of the *AmberTools20*^49^ MD package. The root mean square deviation (RMSD), the root mean square fluctuations (RMSF), the radius of gyration (Rg) and the number of hydrogen bonds of the complex of compound and protein were calculated to determine the stability between each compound and the protein^51^.

### Calculation of binding free energies

The Molecular Mechanics Generalized Born Surface Area (MM-GBSA) method to estimate the binding free energy of RBD with top six compounds and HS by with MMPBSA.py program in *AmberTools20*^49^. The MM-GBSA calculations were applied to 100 snapshots extracted, in the interval of 1 ns, from the 100 ns of the MD simulation of RBD complexes. The MM-GBSA method has the four terms to calculate the free energy, the Van der Waals interaction energy (vdW), the electrostatic energy (ele), the polar solvation energy (pol) and the non-polar solvation energy (nonpol). The free energy difference of binding is composed of the following terms:

$$\Delta {\text{G }} = \, \Delta {\text{E}}_{{({\text{vdW}})}} + \, \Delta {\text{E}}_{{({\text{ele}})}} + \, \Delta {\text{E}}_{{({\text{pol}})}} + \, \Delta {\text{E}}_{{({\text{nonpol}})}}$$

To calculated the binding free energy for each system of RBD complex. The average free energy of complex, receptor and compound were obtained to estimate of the binding free energy as following formula.

$$\Delta {\text{G}}_{{({\text{Binding}})}} = \, \Delta {\text{G}}_{{({\text{Complex}})}} - \, \Delta {\text{G}}_{{({\text{Receptor}})}} - \, \Delta {\text{G}}_{{({\text{Compound}})}}$$

The parameters of MM-GBSA calculations are following the standard MM-PB/GBSA method implemented in the *AmberTools20*^49^.

In our approach, we have chosen to neglect entropic contributions, aligning with the recommendation to avoid entropic calculations in normal mode analysis (NMA) due to their suggested high uncertainties^52–54^. Yang et al. suggested that including entropic contributions calculated by NMA could potentially worsen predictions in both MM-GBSA and MM-PBSA^55^. Hou et al. indicated the inclusion of the entropy term did not consistently improve prediction accuracy, as observed in cases such as α-thrombin, avidin, and cytochrome C peroxidase^53^. They suggested that, even without considering conformational entropy, MM-PBSA could still achieve satisfactory accuracy in ranking ligand affinities, aligning with conclusions from previous studies.

### Principal component analysis (PCA)

Principal component analysis (PCA) is a standard tool in statistical mechanics used in order to determine the correlated motions of the residues to a set of linearly uncorrelated variables call principal components. The PCA of RBD complexes with top six compounds and HS were done using the ProDy^56^. Each RBD complex generated 100 snapshots at 1 ns intervals over the course of a 100 ns MD simulation. Only the Ca positions were used for the analysis. PC1, PC2, and PC3 which represent the first three principal components.

### Binding free energy landscape (BFEL) analysis

In this study, the Binding Free energy landscape (BFEL) is constructed in a 3-dimensional coordinate system^57^: The x axis and the y axis are the first (PC1) and second (PC2) principal components with the highest eigen values calculated from PCA analysis for HS–RBD and compound–RBD complexes and the z axis is the calculated binding free energy of each binding conformation from MM-GBSA. The xy plane was divided into 100 ×100 mesh grids, and the scatter data of binding free energy were fitted to the grids by using Origin8.

### ADME and toxicity prediction

Absorption, distribution, metabolism, excretion, and toxicity (ADMET) prediction was done to determine the drug-likeness properties of selected compounds. The 3D structures of compounds (ACE, HYP, ISO, CAG, CGA, and ORO) were saved in the smiles format and were uploaded on SWISSADME (Molecular Modeling Group of the SIB (Swiss Institute of Bioinformatics), Lausanne, Switzerland) for the prediction. SWISSADME is a web tool used for the prediction of ADME and the pharmacokinetic properties of a molecule. The predicted outcome includes lipophilicity, water solubility, physicochemical properties, and pharmacokinetics^58^. PROTOX-II is a server that predicts the carcinogenicity, cytotoxicity, and the LD50 value and toxicity class of a question molecule in rodents^59^.

## Results

### The conservation analysis of RBD in the *Coronavirus Sarbecovirus* family

**Figure 1:**
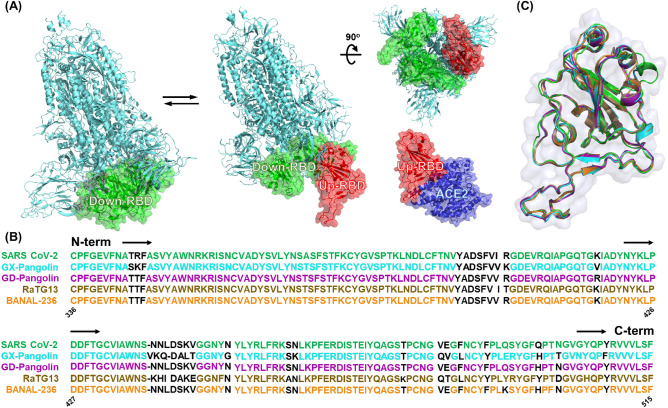
Conservation analysis of receptor binding domain (RBD) in the coronavirus sarbecovirus family. (**A**) The trimeric RBDs consist of the SARS-CoV-2 spike (S) protein. Two conformations of the RBD are presented as down-RBD (green) and up-RBD (red). (**B**) Multiple sequence alignment of SARS-CoV-2 (green); GX-pangolin (cyan); GD-pangolin (purple); RaTG13 (sand) and BANAL-236 (orange). (**C**) Structural superimposition of the receptor binding domains (RBDs) among the sarbecovirus family. The root mean-square deviation (RMSD) values for the backbone atoms in the structural superimposition with respect to the SARS-CoV-2 RBD are as follows: GX-pangolin: 0.94 Å; GD-pangolin: 0.92 Å; RaTG13: 0.80 Å; BANAL −236: 0.78 Å, respectively.

The coronaviruses spike (S) protein plays a key role in receptor recognition and cell membrane fusion. The structure of the S protein is trimeric and consists of a signal peptide (residues 1–13) at the N-terminus, the S1 domain (residues 14–685), and the S2 domain (residues 686–1273). The receptor binding domain (RBD) is located at the bottom of the S1 domain (residues 319–541) and is responsible for receptor binding and membrane fusion (Fig. 1A). The RBD adopted two distinct interconverted conformations, Up-RBD (green) and Down-RBD (red). The Up-RBD configuration is necessary for efficient binding to the human ACE2 receptor^60^.

To analyze the conservation of RBD in the *Sarbecovirius* family, a multiple sequence alignment was performed in the following *Sarbecovirus* RBD, the host including pangolin (GX-pangolin and GD-pangolin), bat (RaTG13 and BANAL-236), and human (SARS-CoV-2). As shown in Fig. 1B, the region of RBD (residues 336–515) in the *Sarbecovirus* has found to be a highly conserved regions among most of the species. Moreover, the superposition of the five RBDs in the *Sarbecovirus* family showed a root-mean-square deviation (RMSD) of the backbone atoms of about 0.78 to 0.94 Å (Fig. 1C). Interestingly, the similar RBD structures consisted of a five-stranded antiparallel β-sheet sandwiched between α-helices and loops arranged in a right-handed, fist-shaped structure conserved among the *Sarbecovirus* family.

### The highly conserved region of RBD in the SARS-CoV-2 variants

**Figure 2:**
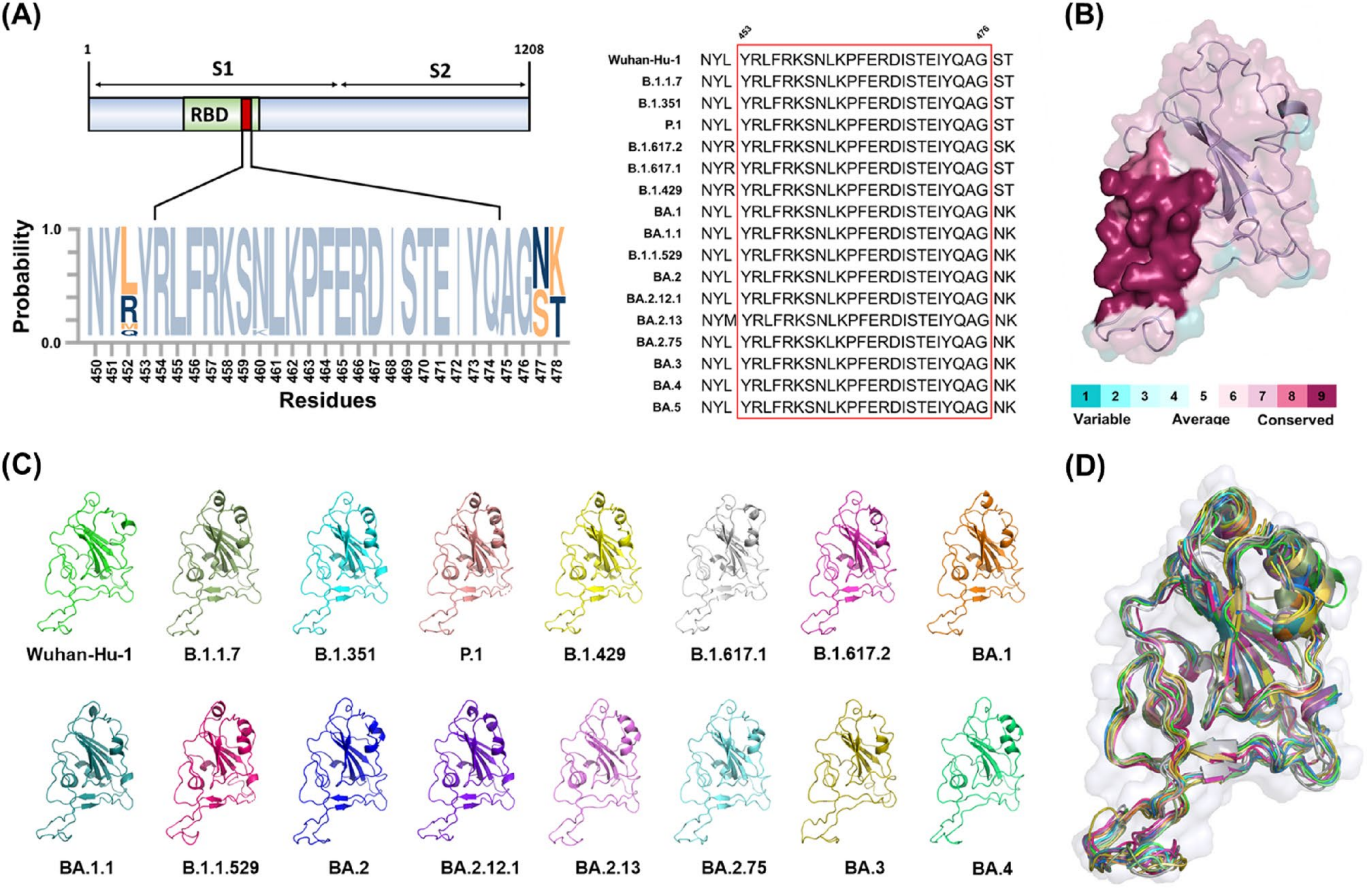
Conservation analysis of receptor binding domains (RBDs) in different SARS-CoV-2 variants. (**A**) Multiple sequence alignment of the different SARS-CoV-2 RBD variants. The red box indicates highly conserved residues (Y453 to G476). (**B**) The surface representation of the RBDs colored according to the level of sequence conservation in the SARS-CoV-2 variants. (**C**) The overall structure of the RBDs in the SARS-CoV-2 variants is depicted as a cartoon and colored as following: Wuhan-Hu-1 (green), B.1.1.7 (lime); B.1.351 (cyan), P.1(salmon), B.1.429 (yellow), B.1.617.1 (gray), B.1.617.2 (light magenta), BA.1 (orange), BA.1.1 (deep teal), B.1.1.529 (hot pink), BA.2 (blue), BA.2.12.1 (purple), BA.2.13 (violet), BA.2.75 (marine), BA.3 (olive), BA.4 (lime green), respectively. (**D**) The structure superimposition of the RBDs structures including the SARS-CoV-2 wild type and the fifteen variants. The RMSD values were showed in Table 1.

Sequence analysis is the process of examining amino acid sequences to understand their features, function, structure, or evolution^61^. To identify the highly-conserved region of the RBD, multiple sequence alignment was performed and analyzed among the wild type (WT; Wuhan-Hu-1) and several SARS-CoV-2 variants, including alpha (B.1.1.7), beta (B.1.351), gamma (P.1), epsilon (B.1.429), kappa (B.1.617.1), delta (B.1.617.2), and omicron (BA.1, BA.1.1, B.1.1.529, BA.2, BA.2.12.1, BA.2.13, BA.2.75, BA.3, BA.4). Interestingly, twenty-four amino acid residues (Y453 to G476) were completely identical in all variants of SARS-CoV-2 RBDs (Fig. 2A). Multi-sequence alignments using the conservation function of *PyMOL* showed that the region (Y453 to G476) was highly conserved between different SARS virus strains (Fig. 2B).

To compare the differences between the shapes in the SARS-CoV-2 S RBD, the structure coordinates of the original and the six RBD variants were obtained from the protein data bank (RCSB PDB) (Fig. 2C). As shown in the Fig. 2D, the superposition of the RBD variants of SARS-CoV-2 showed the RMSD of the backbone atoms of about 0.7 to 1.3 Å, indicating that the structures of the RBD variants of SARS-CoV-2 have a high degree of similarity. The RMSD of the common prevalent delta (B.1.617.2) and omicron (B.1.1.529) variants are 0.9 and 1.1 Å, respectively (Table 1). These results indicated the structural features of the RBD variants in SARS-CoV-2 are highly similar and conserved within the region spanning Y453 to G476 residues.

**Table 1:** The RMSD (Å) results of the structural superimposition between the individual RBDs of SARS-CoV-2 variants.

	Wuhan-Hu-1	B.1.1.7	B.1.351	P.1	B.1.429	B.1.617.1	B.1.617.2	BA.1	BA.1.1
Wuhan-Hu-1	0.0	1.1	0.7	0.9	0.7	1.3	1.0	0.9	0.8
B.1.1.7	1.1	0.0	0.6	0.8	0.7	1.5	0.8	0.7	0.7
B.1.351	0.7	0.6	0.0	0.6	0.4	1.5	0.8	0.5	0.3
P.1	0.9	0.8	0.6	0.0	0.6	1.5	0.8	0.6	0.6
B.1.429	0.7	0.7	0.4	0.6	0.0	1.5	0.8	0.4	0.3
B.1.617.1	1.3	1.5	1.5	1.5	1.5	0.0	1.3	1.4	1.3
B.1.617.2	1.0	0.8	0.8	0.8	0.8	1.3	0.0	0.9	0.9
BA.1	0.9	0.7	0.5	0.6	0.4	1.4	0.9	0.0	0.5
BA.1.1	0.8	0.7	0.3	0.6	0.3	1.3	0.9	0.5	0.0
B.1.1.529	1.3	1.3	1.2	1.2	1.1	1.7	1.3	1.1	1.1
BA.2	1.1	1.3	0.9	0.9	0.9	1.5	1.0	0.8	1.0
BA.2.12.1	0.7	0.8	0.4	0.6	0.4	1.4	0.8	0.4	0.4
BA.2.13	1.2	1.0	1.0	1.0	1.0	1.6	1.1	1.0	1.1
BA.2.75	1.0	0.9	0.8	0.9	0.9	1.3	0.9	0.8	1.0
BA.3	1.5	1.2	1.2	1.2	1.1	1.6	1.9	1.2	1.3
BA.4	1.7	1.6	1.6	1.6	1.6	1.6	1.4	1.5	1.6

	B.1.1.529	BA.2	BA.2.12.1	BA.2.13	BA.2.75	BA.3	BA.4		
Wuhan-Hu-1	1.3	1.1	0.7	1.2	1.0	1.5	1.7		
B.1.1.7	1.3	0.9	0.8	1.0	0.9	1.2	1.6		
B.1.351	1.2	0.9	0.4	1.0	0.8	1.2	1.6		
P.1	1.2	0.9	0.6	1.0	0.9	1.2	1.6		
B.1.429	1.1	0.9	0.4	1.0	0.9	1.1	1.6		
B.1.617.1	1.7	1.5	1.4	1.6	1.3	1.6	1.6		
B.1.617.2	1.3	1.0	0.8	1.1	0.9	1.2	1.4		
BA.1	1.1	0.8	0.4	1.0	0.8	1.2	1.5		
BA.1.1	1.1	1.0	0.4	1.1	0.9	1.3	1.6		
B.1.1.529	0.0	1.5	1.1	1.4	1.4	1.7	1.9		
BA.2	1.5	0.0	1.0	0.9	0.9	0.9	1.4		
BA.2.12.1	1.1	1.0	0.0	1.1	1.0	1.2	1.6		
BA.2.13	1.4	0.9	1.1	0.0	0.9	1.1	1.4		
BA.2.75	1.4	0.9	1.0	0.9	0.0	1.0	1.3		
BA.3	1.7	0.9	1.2	1.1	1.0	0.0	1.5		
BA.4	1.9	1.4	1.6	1.4	1.3	1.5	0.0		

### The druggable pocket prediction of RBD in SARS-CoV-2 variants

**Figure 3:**
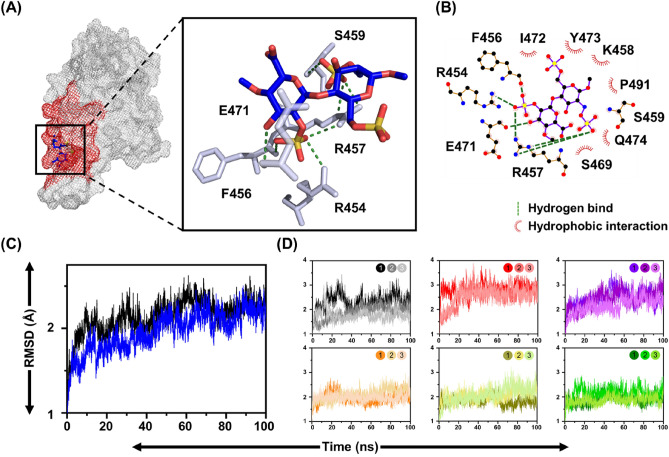
Druggable pocket prediction in RBD of SARS-CoV-2. (**A**) Overall modelled structures of the RBD in complex with heparan sulfate (HS). The RBD was shown in a gray mesh and the binding region in red, while the HS was shown in blue. (**B**) The results of the LigPlot+ analysis revealed the interactive networks of the HS-RBD complex structure. The green and red dashed lines indicated hydrogen bonding and hydrophobic interactions, respectively. (**C**) The RMSD of the RBD (black) and the HS-RBD complex (blue) was shown for the duration of 100 ns. (**D**) The RMSD was calculated using a point mutation simulation method. The results were as follows: wild type (black), R454A (red), E471A (purple), F456A (orange), R457A (dark yellow) and S459A (dark green).

Previous research has shown that the region between Y453 to G476 was the heparan sulfate (HS) binding region in the RBD (HS-RBD binding region)^20^, which was bound by polysaccharide and helped the virus to rapidly enter cells. Blocking this region can affect the life cycle of the virus, and its infection efficiency is greatly reduced^62^. To better understand the details of the binding between HS and RBD, molecular docking with HS and RBD (PDB ID: 7DDD) was performed using *Libdock* module and *Autodock vina,* then further analyzed by LigPlot+ software. As shown in Fig. 3A, HS interacted with SARS-CoV-2 RBD through hydrogen bonding at residues S454, F456, R457, S459, and E471. The hydrophobic interactions with residues K458, S469, I472, Y473, Q474, and P491 indicate that HS interacted closely with RBD (Fig. 3B). To verify the stability of the HS-RBD complex compared to RBD, MD simulation was performed for 100 ns. The average of RMSD was 2.1 ± 0.2 Å for RBD and 2.0 ± 0.3 Å for HS-RBD complex, indicating the HS-RBD complex was more stable than RBD (Fig. 3C) To investigate the importance of the identified residues to HS-RBD binding site, the simulation of point mutations was performed on five residues within hydrogen bonding (R454, F456, R457, S459, and E471) (Fig. 3D). The average RMSD values obtained from the point mutations simulations were 2.0 ± 0.3 Å, 2.6 ± 0.2 Å, 2.3 ± 0.2 Å, 2.0 ± 0.1 Å, 2.0 ± 0.1 Å and 2.0 ± 0.2 Å for the wild type (black), R454A (red), E471A (purple), F456A (orange), R457A (dark yellow) and S459A (dark green), respectively (Fig. S1). The RMSD showed a significantly increasing in the residues R454, and E471 compared to the wild-type, indicated that these two residues play a critical role in the formation of the HS-RBD complex. In addition, binding energy calculations revealed a significant increase in the binding energy of residues R454 and E471 after point mutations, while the other three residues were similar to the wild type (data not shown). These results suggest R454 and E471 play an important role in RBD stability. Consequently, the druggable pocket, including R454, and E471, were considered potential ‘hot-spots’ for further drug development in this study.

### Molecular docking for RBD inhibitors from natural compounds

To detect the possible RBD inhibitors via druggable pocket identified in this study, a molecular docking was performed with the natural compounds database. *LibDock* and *AutoDock vina* were used to screen RBD inhibitors from the NRICM101's herbal ingredients. *LibDock* is a high-throughput algorithm for docking compounds against a receptor's active sites, which in our case were residues of R454, and E471. The compound conformations were docked to polar and apolar receptor interaction sites (hot-spots), and the best-scoring poses were reported. All of the natural products from NRICM101's herbal ingredients (1382 compounds) were docked to the active sites of RBD (PBD ID: 7DDD) with *LibDock*. The resulting 6 compounds most frequently identified in affinity screening (Libdock score > 80) were selected and presented in Table 2. Subsequently, top six candidates were identified according to the Lipinski's rule: (1) the molecular weight of the compounds ranges from 250 to 500 Da, and (2) the ideal LogP ranges from − 2 to 5. (3) more than 3 hydrogen bonds to the receptor SARS-CoV-2 S RBD. In addition, the similar docking pose were also revealed by *Autodock vina* (Fig. S1).

**Table 2:** Chemical details and structures retrieved from the PubChem database.

Compound name	Compound structure	Molecular formula	Molecular weight (g/mol)	Libdock score	Binding energy (kcal/mol)
ACE		C_29_H_36_O_15_	624.60	143.09	** − **234.56
CAG		C_26_H_28_O_13_	548.50	104.44	** − **217.67
HYP		C_21_H_20_O_12_	464.38	92.86	** − **220.32
ISO		C_21_H_20_O_12_	464.38	92.86	** − **220.32
ORO		C_22_H_20_O_11_	460.39	92.49	** − **204.58
CGA		C_26_H_28_O_13_	548.50	88.81	** − **214.03

**Figure 4:**
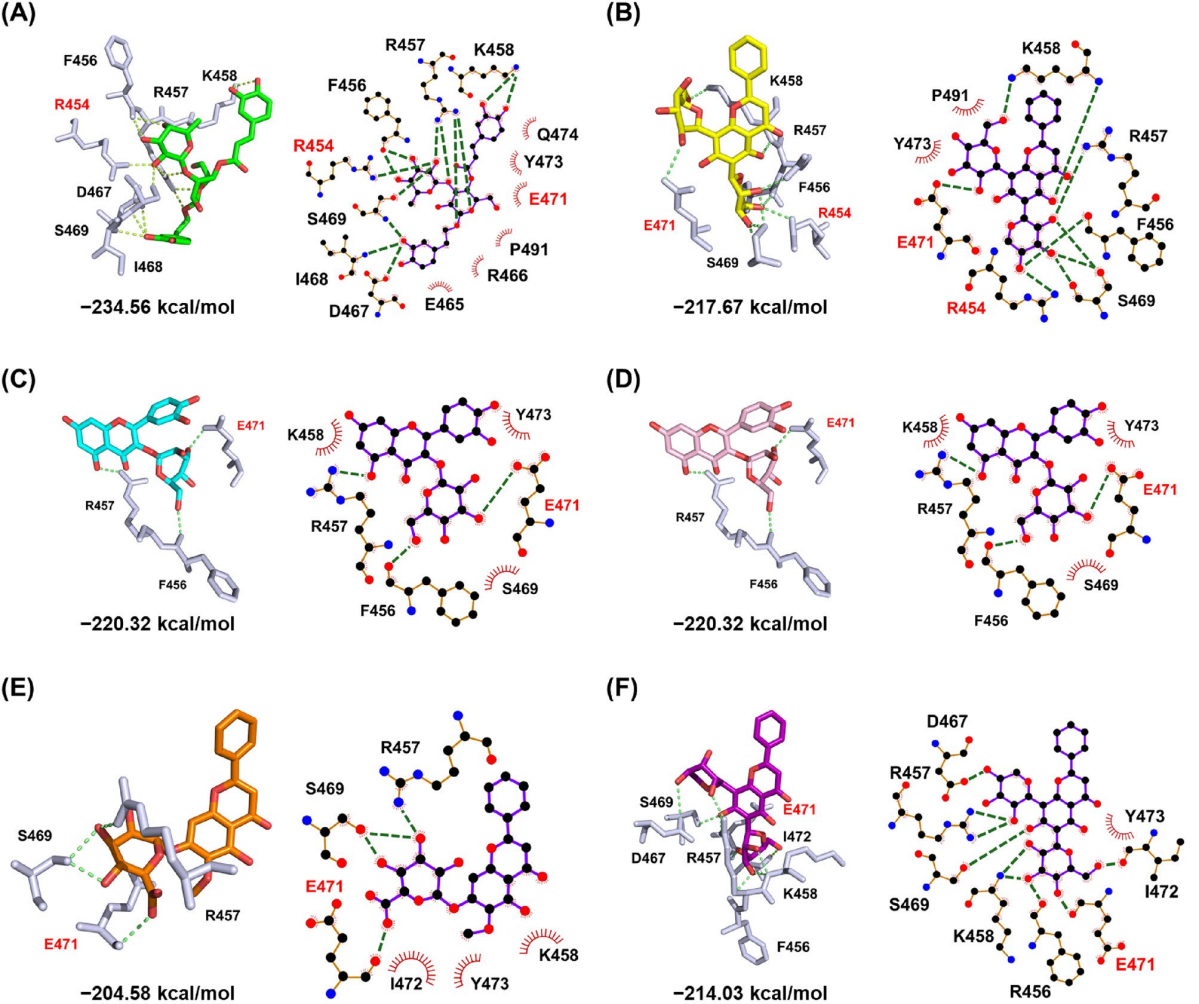
Molecular interactions of RBD with potential inhibitors identified with LibDock. The overall docking poses between RBD and the potential compounds were shown in a stick representation. The results of the LigPlot+ analysis showed the interactive networks of (**A**) ACE (green), (**B**) CAG (yellow), (**C**) HYP (cyan), (**D**) ISO (pink), I ORO (orange) and (**F**) CGA (purple); the active site residues were shown as white sticks and potential hot-spots were highlighted in red. The green dashed lines indicated hydrogen-bonded interactions, while hydrophobic interactions were shown in red.

The molecular interactions and binding energy analysis in the binding pocket of the RBD with the candidate compounds were shown in Fig. 4 and Table 3. The active site of the SARS-CoV-2 RBD exhibited hydrogen bonding with the druggable pocket residues. By looking into the hydrophobic interactions located in residues S469, Y473, and P491, the site of action in the ACE-RBD complex was found to the maximum hydrogen bonds to RBD (R454, F456, R457, K458, D467, I468, and S469) with the lowest binding energy of − 234.56 kcal/mol in *Libdock*. Besides, natural compounds, ISO and HYP, with the same binding energy of − 220.32 kcal/mol in *Libdock*.*,* also illustrated the binding with active site in H-bond and hydrophobic interaction through R456, F457, K458, S469, E471 and Y473. These results revealing the natural compounds, ACE, HYP and ISO can be considered as a potential RBD inhibitor.

**Table 3:** Detail interactions for potential compounds with SARS-CoV-2 RBD.

Compound name	Interaction				LogP
	H-bond		Bond length (Å)	Hydrophobic interaction	
ACE	O40-NH2	R454	2.49	E465, R466, E471, Y473, Q474, P491	− 1.02
	O42-O	F456	3.26		
	O44-O	F456	3.11		
	O5-NH1	R457	2.78		
	O10-NH2	R457	2.87		
	O21-NH2	R457	2.87		
	O40-NH1	R457	2.51		
	O35-NZ	K458	2.75		
	O37-NZ	K458	2.55		
	O19-OD1	D467	3.02		
	O19-N	I468	2.72		
	O19-N	S469	3.01		
	O40-OG	S469	2.57		
CAG	O21-NH2	R454	2.80	Y473, P491	− 1.46
	O21-O	F456	2.79		
	O25-NH1	R457	2.43		
	O15-N	K458	3.21		
	O33-NZ	K458	2.93		
	O23-O	S469	2.82		
	O23-OG	S469	2.62		
	O25-OG	S469	3.30		
	O39-OE2	E471	3.31		
HYP	O9-O	R456	2.75	K458, S469, Y473	− 0.54
	O32-O	F457	2.70		
	O13-OE1	E471	2.68		
ISO	O9-O	R456	2.75	K458, S469, Y473	− 0.54
	O32-O	F457	2.70		
	O13-OE1	E471	2.68		
ORO	O15-NH1	R457	2.76	K458, I472, Y473	0.45
	O15-OG	R469	2.65		
	O13-OG	R469	2.49		
	O11-O	E471	2.81		
CGA	O25-O	F456	3.01	Y473	− 1.46
	O29-NH1	R457	2.59		
	O39-NH2	R457	2.75		
	O25-N	K458	2.90		
	O27-N	K458	2.80		
	O35-OD1	D467	3.32		
	O29-OC	S469	2.56		
	O23-O	E471	2.82		
	O21-O	I472	2.63		

### MD simulation analysis with candidate natural compounds

**Figure 5:**
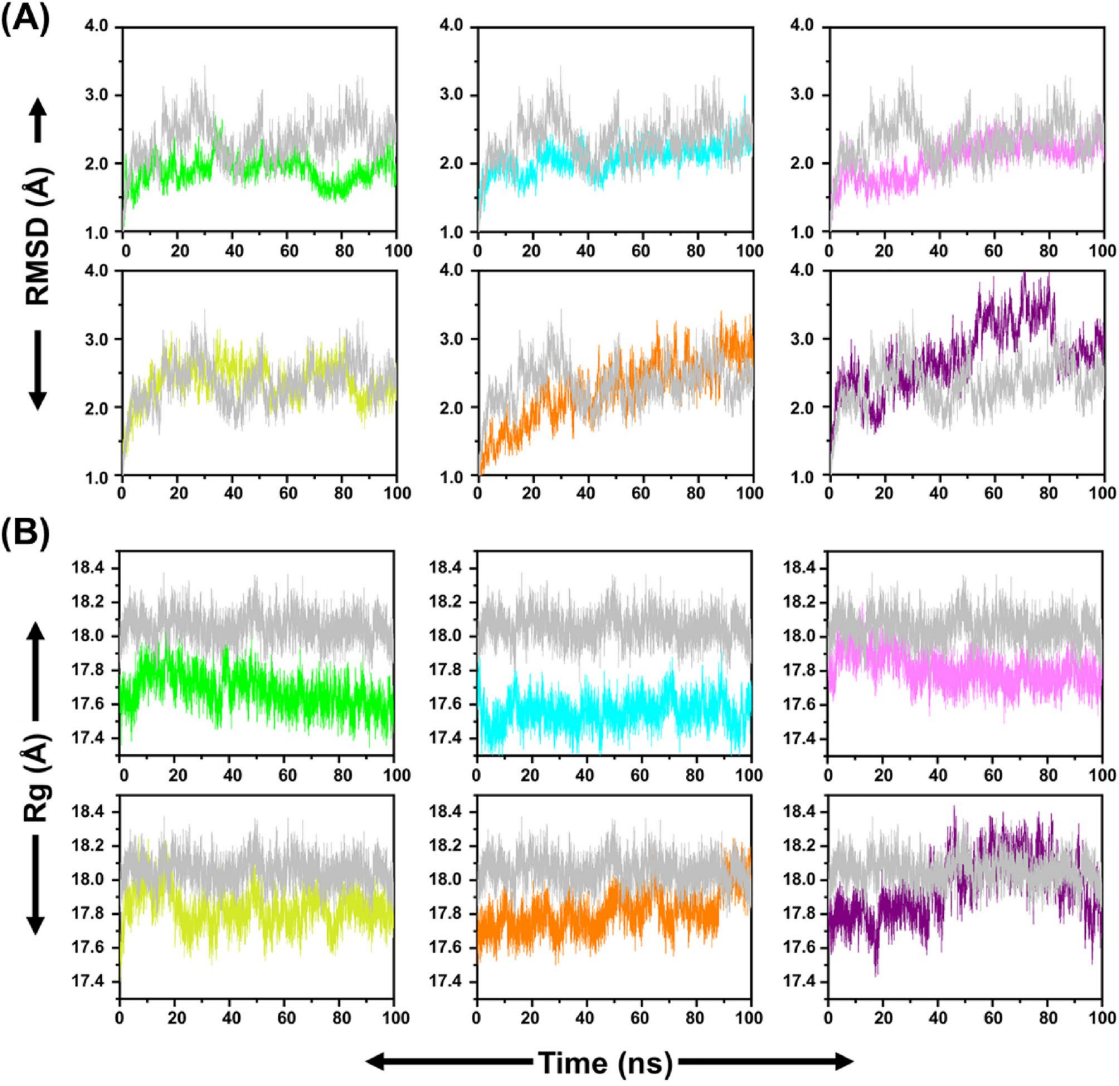
RMSD and Rg analysis of the chain of SARS-CoV-2 RBD showed after binding with HS (gray) and potential compounds ACE (green), ISO (pink), HYP (cyan), CAG (yellow), ORO (orange) and CGA (purple).

To gain insight into the dynamic behavior of the compounds at the active site of the RBD protein, MD simulations were performed for the RBD protein structure and complexed with six candidate compounds (Fig. 5A). To quantify the structural stability of the protein-compound complexes, the root-mean-square deviation (RMSD) of the backbone Cα atoms was measured. The average of RMSD was reported 2.3 ± 0.3 Å for HS-RBD. However, the average of RMSD for RBD complexed with ACE, HYP, ISO, CAG, ORO, and CGA were reported as 1.9 ± 0.2 Å, 2.1 ± 0.2 Å, 2.1 ± 0.3 Å, 2.4 ± 0.3 Å, 2.2 ± 0.5 Å and 2.7 ± 0.5 Å, respectively (Fig. S2. These results indicated that the docked poses of candidates in the MD simulation were reliable under conditions closed to well-equilibrated systems.

Radius of gyration (Rg) is a parameter linked to the tertiary structural volume of a protein-compound complex and has been applied to obtain insight into the stability of a protein-compound complex in a biological system. A complex is supposed to have a higher radius of gyration due to less tight packing. The average Rg values for HS, ACE, HYP, ISO, CAG, ORO, CGA were found to be 18.0 ± 0.1 Å, 17.7 ± 0.1 Å, 17.7 ± 0.2 Å, 17.8 ± 0.1 Å, 17.8 ± 0.1 Å, 17.8 ± 0.1 Å, and 17.8 ± 0.1 Å, respectively (Fig. S3). Based on the plots of Rg (Fig. 5B), the curve of HS was positioned above those of three complexes, ACE, HYP, and ISO. These results suggested that the overall stability of the protein-compound complex in these three complexes were better than HS.

### Analyzing the communication between RBD and candidate natural compounds

**Figure 6:**
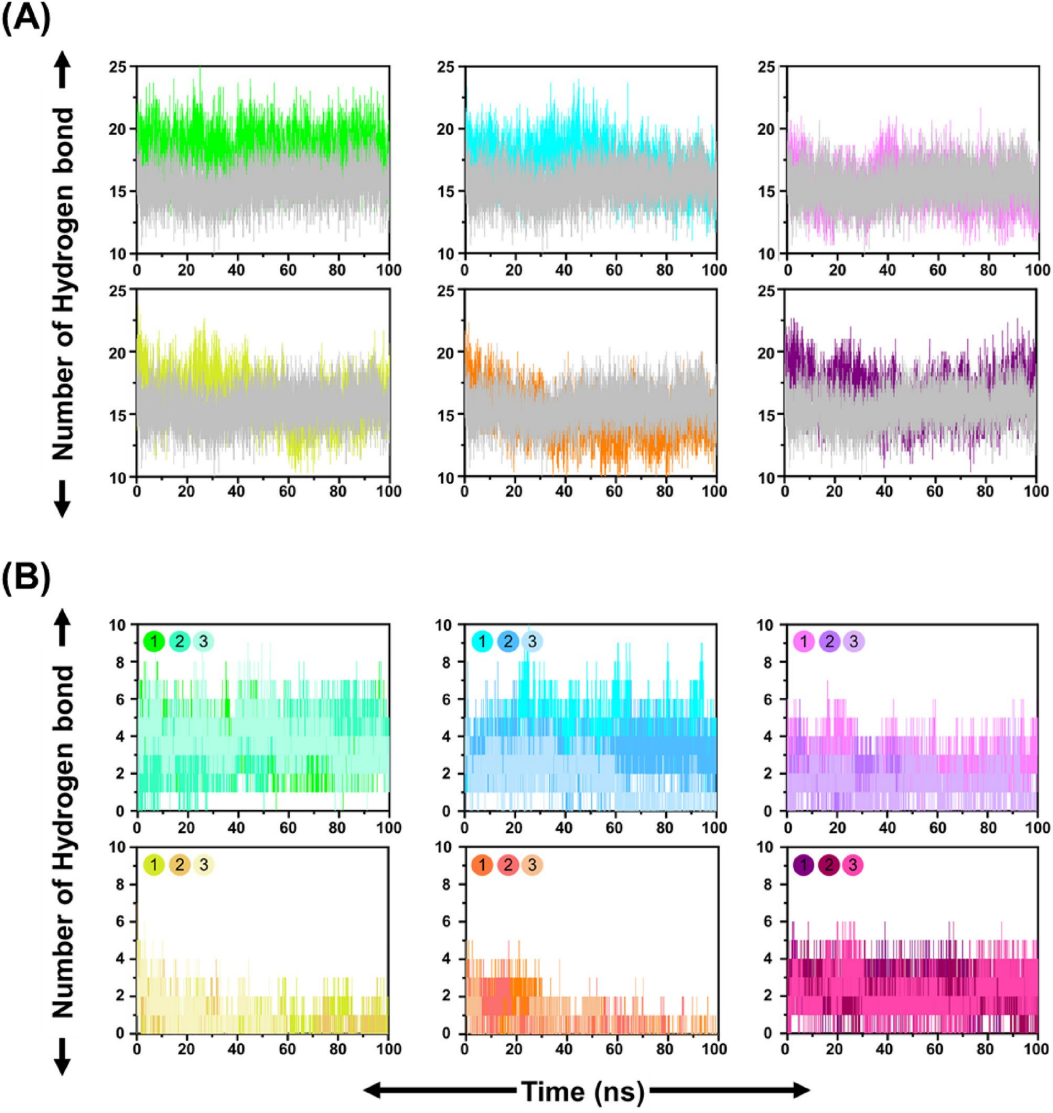
Time-dependent analysis of the number of hydrogen bonds (number of H-bonds) for (**A**) total complex or (**B**) only contributed by compounds. ACE-RBD (green), HYP-RBD (cyan), ISO-RBD (pink), CAG-RBD (yellow), ORO-RBD (orange) and CGA-RBD (purple), and HS-RBD (gray).

The interaction between the top six potential compounds was further preformed the hydrogen bonding analysis and PCA. The average number of hydrogen bonds of RBD complexed with ACE, HYP, ISO, CAG, ORO, and, CGA were calculated as 18.2 ± 1.6, 17.0 ± 1.7, 15.4 ± 1.6, 16.2 ± 1.8, 14.7 ± 1.8 and 16.5 ± 1.7 Å respectively (Fig. 6). Compared to the reference natural HS complexed RBD were calculated as 15.5 ± 1.4 hydrogen bonds formation (Fig. 6A). These results were consistent with the RMSD of RBD complexed with candidates for MD simulation (Fig. 5). In the Fig. 6B, four compounds, including ACE, ISO, HYP, and CGA, revealed strong hydrogen bonding with the protein through the course of simulation. It can be seen that ACE has the highest number of hydrogen bonds, down to which the second-highest is compound ISO and then HYP. Hydrogen bonding in the case of CAG, and ORO is lower than the rest of the compounds.

**Figure 7:**
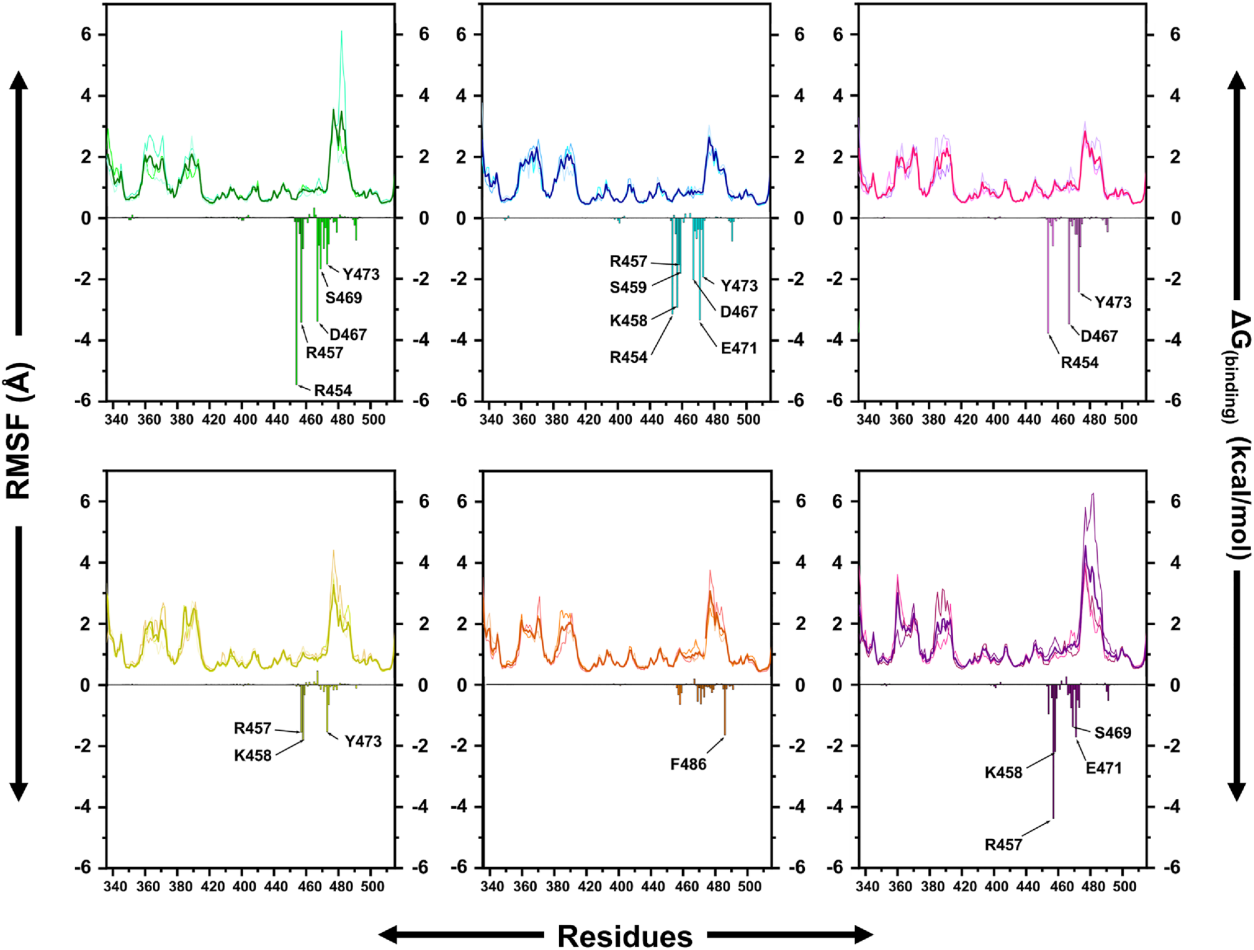
RMSF values extracted from protein ft ligand of the protein–ligand docked complexes including ACE-RBD (green), HYP-RBD (cyan), ISO-RBD (pink), CAG-RBD (yellow), ORO-RBD (orange) and CGA-RBD (purple). Decomposition of binding free energy on a per-residue basis for each compound-RBD complex. The unit for energy contribution per residue is kcal mol^−1^.

Next, we compared the root mean square fluctuations (RMSF) of C-α atoms during the MD for each compound-RBD complex. While the RMSF values in the case of all six compounds were very similar, the fluctuations in the CGA were considerably higher. Notably, the RMSF values at the active sites around (R454-E471) remain consistently low as well as the results shown in triplicate of simulation. These findings were confirmed by residue-based free energy decomposition analysis (Fig. 7). Generally, if the ΔG_(Binding)_ interaction energy calculated by MM-GBSA, between the residue and the substrate is lower than − 1 kcal mol^−1^, comprised of ΔE _(vdW)_, ΔE _(Elec)_, ΔE _(Polar Solv)_ and ΔE _(Non-polar Solv)_, those residues are considered to be important in potential hot-spots (Table 4).

**Table 4:** Binding energy contribution per residue from MM-GBSA calculation.

	Residue	ΔE_(vdW)_	ΔE_(Elec)_	ΔE_(Polar Solv)_	ΔE_(Non-polar Solv)_	ΔG_(Binding)_
ACE	R454	− 0.6 ± 0.5	− 6.4 ± 2.7	1.6 ± 1.7	0.0 ± 0.0	− 5.5 ± 1.3
	R457	− 2.6 ± 0.6	− 1.0 ± 1.9	0.4 ± 1.9	− 0.3 ± 0.1	− 3.4 ± 1.6
	D467	0.5 ± 1.2	− 17.1 ± 5.7	13.4 ± 3.4	− 0.2 ± 0.0	− 3.4 ± 2.0
	S469	− 1.9 ± 0.4	1.3 ± 1.2	− 0.8 ± 0.8	− 0.2 ± 0.0	− 1.7 ± 0.9
	Y473	− 1.6 ± 0.5	− 0.2 ± 0.4	0.5 ± 0.3	− 0.2 ± 0.1	− 1.5 ± 0.6
HYP	R454	− 0.7 ± 0.5	− 2.5 ± 2.2	0.1 ± 1.7	0.0 ± 0.0	− 3.1 ± 0.8
	R457	− 3.1 ± 0.5	− 3.4 ± 2.6	3.9 ± 2.3	− 0.3 ± 0.1	− 2.9 ± 1.0
	K458	− 1.4 ± 0.8	− 5.5 ± 2.0	5.6 ± 2.0	− 0.2 ± 0.1	− 1.5 ± 0.6
	S459	− 0.1 ± 0.7	− 2.4 ± 1.8	0.8 ± 0.6	− 0.1 ± 0.0	− 1.8 ± 1.0
	D467	− 0.8 ± 1.1	− 13.8 ± 1.7	11.1 ± 2.6	− 0.1 ± 0.0	− 2.0 ± 1.4
	E471	− 0.6 ± 1.0	− 8.6 ± 6.7	6.1 ± 5.3	− 0.3 ± 0.1	− 3.3 ± 1.6
	Y473	− 1.5 ± 0.3	− 1.1 ± 0.6	0.8 ± 0.4	− 0.1 ± 0.1	− 1.9 ± 0.5
ISO	R454	− 0.3 ± 0.6	− 5.7 ± 2.7	2.4 ± 1.9	− 0.1 ± 0.0	− 3.8 ± 1.2
	D467	1.3 ± 1.0	− 14.0 ± 3.0	9.3 ± 2.1	− 0.1 ± 0.0	− 3.5 ± 1.7
	Y473	− 2.9 ± 1.1	− 0.4 ± 0.6	1.3 ± 0.9	− 0.4 ± 0.1	− 2.4 ± 0.8
CAG	R457	− 1.2 ± 1.1	− 5.2 ± 3.7	5.0 ± 3.4	− 0.2 ± 0.2	− 1.6 ± 1.7
	K458	− 1.9 ± 1.2	− 7.4 ± 5.3	7.8 ± 5.2	− 0.4 ± 0.2	− 1.8 ± 1.7
	Y473	− 1.9 ± 1.3	− 0.5 ± 0.9	1.2 ± 1.4	− 0.3 ± 0.2	− 1.5 ± 1.1
ORO	F486	− 1.7 ± 2.1	− 0.2 ± 0.5	0.5 ± 0.7	− 0.3 ± 0.4	− 1.6 ± 2.1
CGA	R457	− 2.4 ± 1.2	− 6.6 ± 4.9	5.0 ± 3.7	− 0.3 ± 0.1	− 4.4 ± 1.9
	K458	− 2.1 ± 1.6	− 8.0 ± 4.8	8.3 ± 5.0	− 0.4 ± 0.3	− 2.2 ± 1.7
	S469	− 1.0 ± 0.7	− 0.4 ± 1.7	0.2 ± 0.9	− 0.2 ± 0.1	− 1.4 ± 0.7
	E471	− 0.4 ± 0.9	− 6.6 ± 4.5	5.4 ± 3.1	− 0.2 ± 0.1	− 1.7 ± 1.5

Notably, HYP exhibited the most hydrogen bonding interacting with RBD. However, the overall stability of the compound-protein complex was affected by various factors containing electrostatic, hydrogen bonding, van der Waals, and solvation forces, etc. As shown in Fig. S4, the binding free energy by solvation for HYP-RBD was the most unstable. Considering all contributing factors, the stability of ACE-RBD still outperformed that of HYP-RBD.

### Essential dynamics PCA analysis for candidate natural compounds

**Figure 8:**
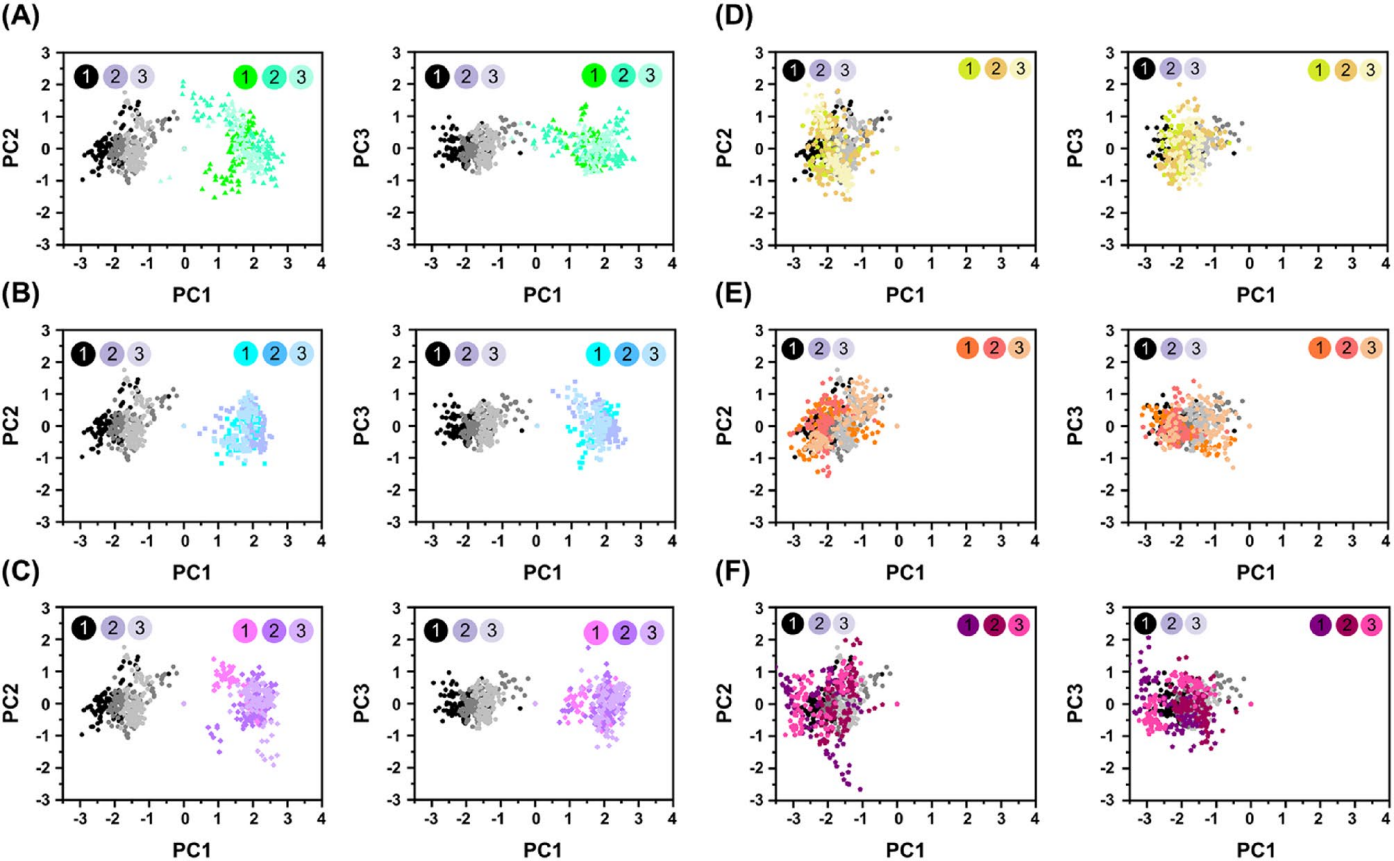
Principal component analysis for complexes between HS-RBD (black) and (**A**) ACE-RBD (green), (**B**) HYP-RBD (cyan), (**C**) ISO-RBD (pink), (**D**) CAG-RBD (yellow), I ORO-RBD (orange), and (**F**) CGA-RBD (purple). The entire simulation trajectory for each compound-RBD complex was used to plot the PCA for extracting the principal motions information about the conformational status. The percentage of total mean square displacement of residue positional variations recorded in each dimension is categorized by equivalent eigenvalue (PCs).

In order to understand the principal structural dynamics revealed by each compound-RBD complex, we plotted the PCA analysis. The given figures showed three eigenvectors or PCA for the RBD being docked with the top six potential compounds based on their extracted trajectories and exposed in clusters. Examining these eigenvectors supports the solid and clustered motions in the target’s corresponding complexes during the simulation. All the compounds exhibited a distinct transition at various points, indicating the alteration in conformation caused by the attachment of the compound. Each dot represents an individual frame. Importantly, ACE, ISO, and HYP in Fig. 8 exhibited distinct dynamic trends upon binding to RBD in comparison to HS. These results suggested that these compounds had the potential to block the interaction between RBD and HS without being utilized by RBD in infecting host cells. Conversely, the PCA distributions of the other compounds, CAG, ORO, and CGA, complexed to RBD are similar to that of HS, suggesting the possibility of downstream effects similar to HS-RBD interactions. Furthermore, local hydrogen bonding analysis (Fig. 6B) revealed CAG, ORO, and CGA compounds exhibited few hydrogen bonds in the MD, indicating a reduced capacity to stabilize complexes. These findings suggested ACE, HYP, and ISO may have more potential in combating SARS-CoV-2.

### Identification of key residues involved in compound binding to RBD from binding free energy landscape analysis

**Figure 9:**
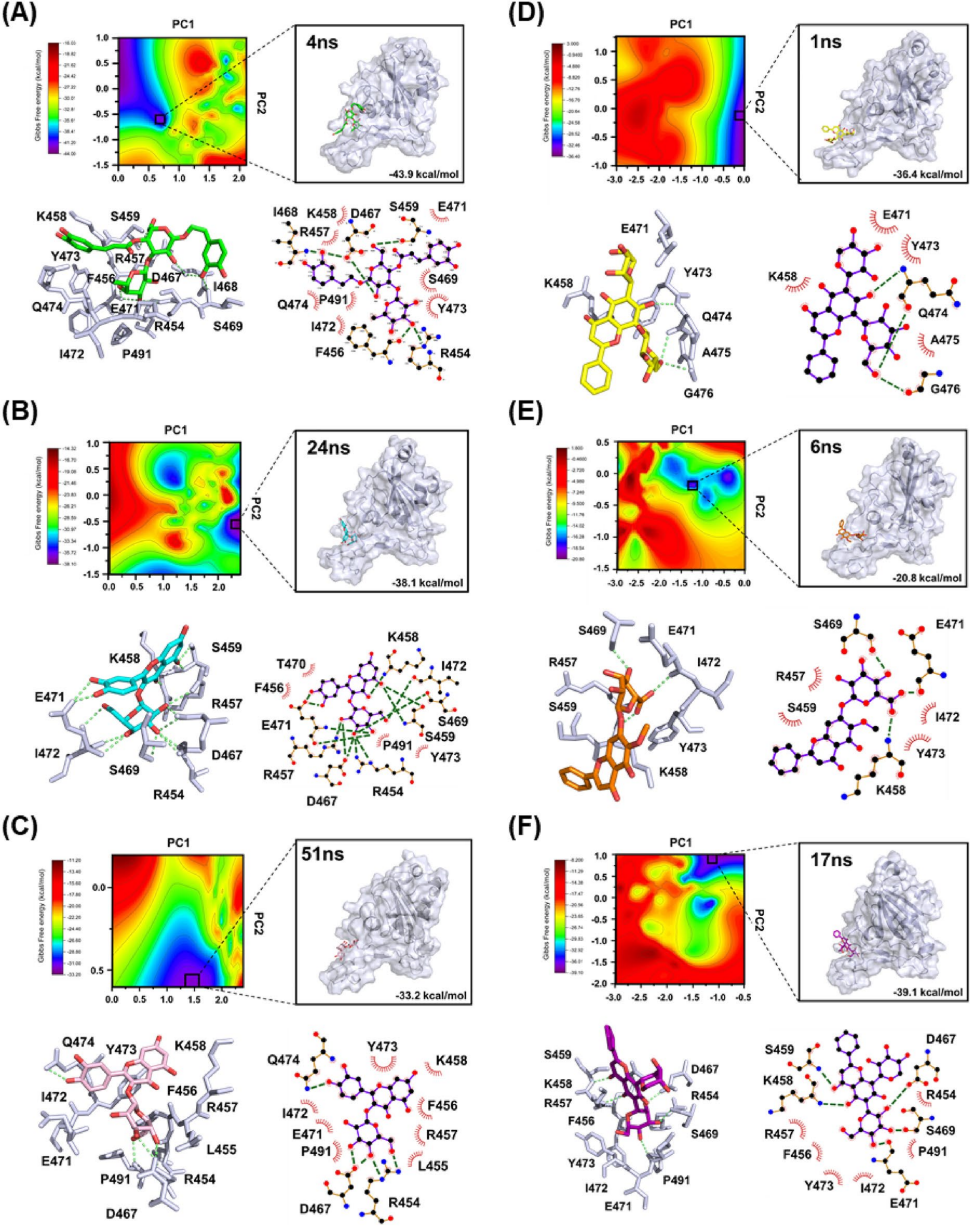
Binding Free energy landscape analysis and representative snapshots extracted from the MD trajectory. Areas in dark blue possess lower energy than other areas. One representative structures of most populated clusters in (**A**) ACE-RBD (green), (**B**) HYP-RBD (cyan), (**C**) ISO-RBD (pink), (**D**) CAG-RBD (yellow), I ORO-RBD (orange), and (**F**) CGA-RBD (purple) complexes.

Then, the lowest energy conformation, calculated by MM-GBSA, was selected in the free energy surface from the most abundant clusters as a part of stable low-energy conformation for the analysis object. Binding Free-energy landscape (BFEL) was used to obtain several low-energy conformations, which are the most suitable analytical subjects according to RMSD-equalized trajectory (Fig. 9). The ACE-RBD docked complex has the most stable dynamics structure since its relative stability of binding free energy was − 43.9 kcal/mol while having six hydrogen bonds with eight hydrophobic interactions (Fig. 9A). The HYP-RBD relative stability of binding free energy was − 38.1 kcal/mol, thus ranking it the second most stable complex with fourteen hydrogen bonds and four hydrophobic interactions (Fig. 9B). The third most stable docking complex is ISO-RBD, with relative stability of binding free energy of − 33.2 kcal/mol, forming five hydrogen bonds with eight hydrophobic interactions (Fig. 9C). Importantly, the residues such as R454 and E471 were all observed to participate in forming stable conformations.

### ADME and toxicity prediction of candidate natural compounds

The ADME properties of the selected natural compounds predicted by SwissADME are summarized in Table 5. All the natural compounds exhibit Log Po/w values below 5, indicating favorable lipophilicity characteristics that may confer protection against ROS degradation. The TPSA (topological polar surface area) ranges from 176.12 to 245.29 Å^2^, with consensus log Po/w values falling within the optimal range of − 1.26 to 0.55. Compounds satisfying the TPSA ≤ 250 Å^2^ parameter are expected to display adequate solubility, oral bioavailability, and cell permeability. With regards to intestinal absorption (human), absorbance of less than 30% is considered to be poorly absorbed. HYP and ISO were predicted to have a poor absorption. P-glycoprotein is a member of the ATP-binding transmembrane glycoprotein family [ATP-binding cassette (ABC)], which can excrete drugs or other exogenous chemicals from cells. The results suggested that ACE, CAG, CGA, and Oroxyloside are substrates of P-glycoprotein—they may be actively exuded from cells by P-glycoprotein. Cytochrome P450s is an important enzyme system for drug metabolism in liver. The two main subtypes of cytochrome P450 are CYP2D6 and CYP3A4. The results showed that all compounds were not substrates for the two subtypes. The ProTox-II Web server was employed to assess organ toxicity profiles, toxicological endpoints, and LD50 values of the compounds. As shown in Table 6, all selected natural compounds are non-carcinogenic and non-cytotoxic, indicating their safety for use.

**Table 5:** ADME properties of potential compound predicted by SwissADME.

Compounds	ACE	HYP	ISO	CAG	ORO	CGA
Physicochemical properties						
Formula	C_29_H_36_O_15_	C_21_H_20_O_12_	C_21_H_20_O_12_	C_26_H_28_O_13_	C_22_H_20_O_11_	C_26_H_28_O_13_
Num. heavy atoms	44	33	33	39	33	39
Num. arom. heavy atoms	12	16	16	16	16	16
Fraction Csp3	0.48	0.29	0.29	0.42	0.27	0.42
Num. rotatable bonds	11	4	4	4	5	4
Num. H-bond acceptors	15	12	12	13	11	13
Num. H-bond donors	9	8	8	9	5	9
Molar refractivity	148.42	110.16	110.16	131.24	111.19	131.24
TPSA	245.29 Å^2^	210.51 Å^2^	210.51 Å^2^	230.74 Å^2^	176.12 Å^2^	230.74 Å^2^
Lipophilicity						
Log *P*_o/w_ (iLOGP)	2.15	1.45	0.94	1.90	1.84	1.97
Log *P*_o/w_ (XLOGP3)	− 0.50	0.36	0.36	− 1.83	1.44	− 1.83
Log *P*_o/w_ (WLOGP)	− 1.12	− 0.54	− 0.54	− 2.11	0.45	− 2.11
Log *P*_o/w_ (MLOGP)	− 2.37	− 2.59	− 2.59	− 3.49	− 1.42	− 3.49
Log *P*_o/w_ (SILICOS-IT)	− 1.14	− 0.59	− 0.59	− 0.85	0.44	− 0.85
Consensus Log *P*_o/w_	− 0.60	− 0.38	− 0.48	− 1.28	0.55	− 1.26
Water solubility						
Log *S* (ESOL)	− 2.87	− 3.04	− 3.04	− 2.13	− 3.63	− 2.13
Log *S* (Ali)	− 4.18	− 4.35	− 4.35	− 2.50	− 4.74	− 2.50
Log *S* (SILICOS-IT)	− 0.22	− 1.51	− 1.51	− 1.31	− 2.91	− 1.31
Pharmacokinetics						
GI absorption	Low	Low	Low	Low	Low	Low
BBB permeant	No	No	No	No	No	No
P-gp substrate	Yes	No	No	Yes	Yes	Yes
CYP1A2 inhibitor	No	No	No	No	No	No
CYP2C19 inhibitor	No	No	No	No	No	No
CYP2C9 inhibitor	No	No	No	No	No	No
CYP2D6 inhibitor	No	No	No	No	No	No
CYP3A4 inhibitor	No	No	No	No	No	No
Log *K*_p_ (skin permeation)	− 10.46 cm/s	− 8.88 cm/s	− 8.88 cm/s	− 10.95 cm/s	− 8.09 cm/s	− 10.95 cm/s

**Table 6:** Toxicity prediction potential compound predicted by ProTox-II.

Endpoint	Target	ACE	HYP	ISO	CAG	ORO	CGA
Organ toxicity	Hepatoxicity	Inactive	Inactive	Inactive	Inactive	Inactive	Inactive
Toxicity end point	Carcinogenicity	Inactive	Inactive	Inactive	Inactive	Inactive	Inactive
	Immunotoxicity	Active	Active	Active	Active	Active	Active
	Mutagenicity	Inactive	Inactive	Inactive	Inactive	Inactive	Inactive
	Cytotoxicity	Inactive	Inactive	Inactive	Inactive	Inactive	Inactive
	LD50 (mg/kg)	5000	5000	5000	536	5000	536
	Toxicity class	5	5	5	4	5	4
Tox21-nuclear receptor signaling pathways	Aryl hydrocarbon receptor (AhR)	Inactive	Inactive	Inactive	Inactive	Inactive	Inactive
	Androgen receptor (AR)	Inactive	Inactive	Inactive	Active	Inactive	Active
Tox21-stress pathways	Heat shock factor response element (HSE)	Inactive	Inactive	Inactive	Inactive	Inactive	Inactive

## Discussion

SARS-CoV-2 is a pandemic respiratory infectious disease with serious public health and economic implications^63^. In view of the serious situation, many antiviral drugs and vaccines have been developed against COVID-19 targeting the SARS-CoV-2 spike (S) protein^64,65^. However, the efficacy of drugs and vaccines is usually limited by multiple spike mutations of the SARS-CoV-2 variants^1,66,67^. To address the challenge of treating SARS-CoV-2 variants, identifying highly conserved sequences and potentially druggable pockets for drug development represents a promising strategy^68^. In SARS-CoV-2 infection, heparan sulfate (HS) from host molecules is essential for recognition of the receptor binding domain (RBD) protein in the SARS-CoV-2 spike by ACE2, regardless of the high variability and therefore prevents the virus from easily acquiring drug resistance^69^. On the other hand, specific viral structural proteins with highly conserved protein sequences, such as the spike RBD are expressed during viral infection.

The spike RBD is located at the bottom of the S1 domain and plays an important role in host ACE2 receptor binding within membrane fusion through HS-assisted^70–72^. In the current study, we observed that RBD is associated with the recognition of ACE2 ability and is also high-conversed in all SARS-CoV-2 variants. The results of sequence alignments showed that the wild type SARS-CoV-2 has highly conserved sequences from Y453 to G476 among other variants such as beta, gamma, delta, kappa, epsilon and omicron. In addition, structural alignments of all SARS-CoV-2 variants revealed that the highly conserved protein sequences were also located in the HS binding region of RBD.

The detail interactions were found that the cellular HS interacted with RBD by forming hydrogen bonds at residues R454, F456, R457, S459 and, E471 and by hydrophobic interactions with residues K458, S469, I472, Y473, Q474 and, P491. Therefore, to block this region as a reasonable target and feasible strategy for the design and development of anti-COVID-19. Notably, the development of antiviral drugs obtained from this approach will be broad-spectrum agents targeting viruses that use the interaction between RBD and HS to facilitate their life cycle, including COVID-19. In drug design, the identification of key residues is important. In the current study, binding positions from HS served as clues, and the simulation of point mutations applied to probe potentially significant sites. The RMSD changes for each residue post-mutation. The analysis of RMSD yielded insights into the impact of point mutations on complex stability. In Fig. 3, significant RMSD elevation was observed for R454A and E471A mutations after a 100 ns simulation, underscoring the pivotal roles of these two residues in complex stability. To avoid the possibility of a high mutation rate in the region where HS binds to RBD, the active sites including R454 and E471 as essential ‘hot-spots’ were further selected for the development of a potent antiviral drug in this study.

Natural products were demonstrated remarkable efficacy against SARS-CoV-2 infection^73–76^. For example, Crocin, a terpenoid compound, was showed a promising binding affinity with the major protease of SARS-CoV-2 in the docking study^77^. Broussochalcone A, a flavonoid isolated from *Broussonetia papyrifera* (L.), has higher affinity and stability in the Mpro of SARS-CoV-2 than lopinavir^78^. Berberine isolated from *Hydrastis canadensis* L. was shown to have a much lower binding energy to chymotrypsin-like protease (3CLpro). The MD simulation of berberine in a complex with 3Clpro showed high stability and indicated a strong effect against SARS-CoV-2 by decreasing the activity of 3Clpro^79^. In this study, the active natural compounds in a traditional herbal formula, NRICM101, were investigated for the treatment of SARS-CoV-2 variants. The molecular docking and MD simulations were performed for several natural compounds from NRICM101 with the highly conserved region of RBD spike protein. These results in the study revealed three potential natural compounds, including Acetoside (ACE), Hyperoside (HYP), and Isoquercitrin (ISO), with a strong affinity to the RBD. These natural compounds were studied for antiviral or pharmacological activities, however, it is the first time to reveal how these natural compounds bind to RBD, leading to their potential as RBD inhibitors against the SARS-CoV-2 variants. In detail, ACE, extracted from *Scutellaria baicalensis* R., exhibited the highest binding affinity to RBD. ACE was also proposed for anti-SARS-CoV-2 effects by inhibiting 3Clpro^80^. HYP, a prominent component of *Abelmoschus odelli* (L.) *medik*, was also identified as having anti-SARS-CoV-2 effects by inhibiting 3Clpro and ACE2 activity^81^. Besides, HYP also showed the anti-cancer effects by mediation of the NF-κB signaling pathway^82^. ISO, derived from *Hypericum perforatum* L., demonstrated anti-inflammatory activity effects by reducing prostaglandin E2 levels^83^*.*

The PCA analysis revealed the dynamic trends of compound complexed to protein.

The PCA results demonstrated distinctions in the binding of natural HS to the RBD protein compared to three potential compounds, ACE, HYP, and ISO. These distinctions implied potential inhibitory behavior for the three compounds, as the movement trends of the complexes significantly differed from the natural HS-RBD complex. Combining with the observed stability of the complex by BFEL, indicating that there these three compounds have the potential as RBD inhibitors to form a stable complex with RBD. While further in vitro and in vivo testing is essential, our initial studies have uncovered the possibility of ACE, HYP, and ISO in treating the *Sarbecovirus* family, including SARS-CoV-2 variants.

## Conclusion

In summary, the bioinformatics and structural information were analyzed and identified the highly conserved regions of RBD in the *Coronavirus Sarbecovirus* family and SARS-CoV-2 variants. Further, the structure-based computational approach with molecular docking and MD simulation was performed to screen and characterize the potential inhibitors of SARS-CoV-2 spike protein RBD. With the establishment of the ‘hot-spots’ model, 1382 natural products from NRICM101 were comprehensively screened, and the compounds ACE, ISO and HYP were identified that apparently interfere with the RBD activities in this study. We demonstrated that ACE blocked the active site of RBD by interacting with residues R454, F456, R457, K458, D467, I468, and S469 via hydrogen bond interactions and hydrophobic contacts with E654, R466, E471, Y473, Q474 and P491, which are key residues for the structure-based lead optimization against RBD protein. The discovery of the RBD inhibitor ACE from *Scutellaria baicalensis R*. holds great potential for the development of new and promising therapeutics for the treatment of SARS-CoV-2 variants.

### Supplementary Information

Supplementary Information.

## Data Availability

All data will be available upon request to the corresponding author.

## Abbreviations

S protein: Spike protein
HS: Heparan sulfate
RBD: Receptor binding domain
RMSD: The root mean square deviation
RMSF: The root mean square fluctuations
ACE: Acetoside
ISO: Isoquercitrin
HYP: Hyperoside
CAG: Chrysin 6-C-arabinoside 8-C-glucoside
ORO: Oroxyloside
CGA: Chrysin 6-C-glucoside 8-C-arabinoside
MD: Molecular dynamics
PCA: Principal component analysis
FEL: Free energy landscape
