# Supplementary material for: Targeting the receptor binding domain and heparan sulfate binding for antiviral drug development against SARS-CoV-2 variants
Source: Sci Rep. 2024 Feb 2;14:2753. doi: 10.1038/s41598-024-53111-2 (PMC10837157; doi:10.1038/s41598-024-53111-2)
Supplement: Supplementary file 1 — Supplementary Information. [file 41598_2024_53111_MOESM1_ESM.docx]

***Targeting the Receptor Binding Domain and Heparan Sulfate Binding for Antiviral Drug Development against SARS-CoV-2 Variants***

Zi-Sin Yang^1,2,†^, Tzong-Shiun Li^3,4,†^, Yu-Sung Huang^2^ , Cheng-Chung Chang^3^, and Ching-Ming Chien^1,^*

1. Department of Medical Sciences Industry, College of Health Sciences, Chang Jung Christian University, Tainan 711, Taiwan
2. Institute of Bioinformatics and Structural Biology, National Tsing Hua University, Hsinchu 300, Taiwan
3. Graduate Institute of Biomedical Engineering, National Chung Hsing University, Taichung 402, Taiwan
4. Department of Plastic Surgery, Show Chwan Memorial Hospital, Changhua 500, Taiwan

† Authors contributed equally to this work

* Corresponding author: [cmchien@mail.cjcu.edu.tw](mailto:cmchien@mail.cjcu.edu.tw)


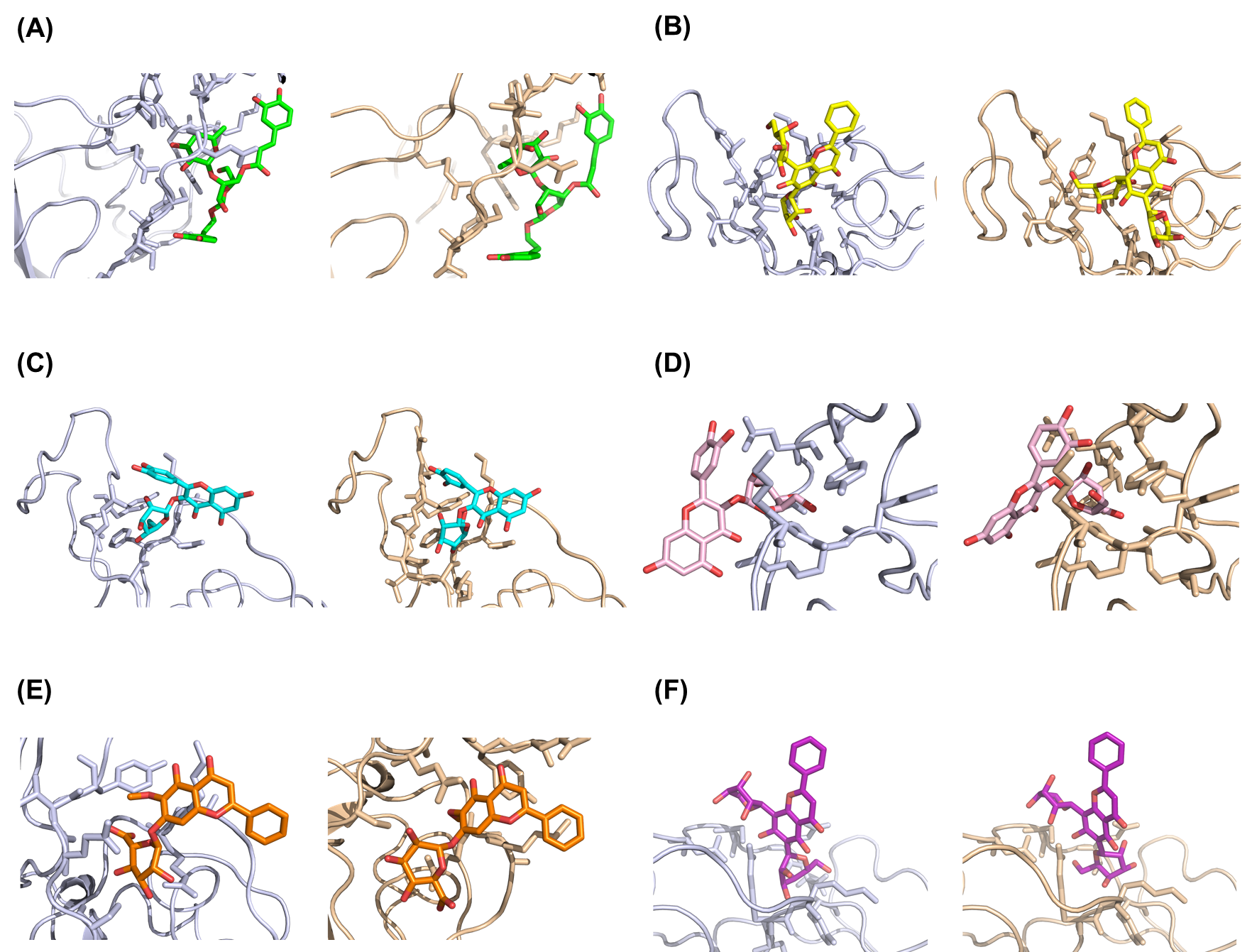


**Figure S1.**  Molecular interactions of RBD with potential inhibitors identified with *Libdock* module (left side) and *Autodock vina* (right side). The overall docking poses between RBD and the potential inhibitors are shown in a stick representation. **(A)** ACE (green), **(B)** CAG (yellow), **(C)** HYP (cyan), **(D)** ISO (pink), **(E)** ORO (orange) and **(F)** CGA (purple)


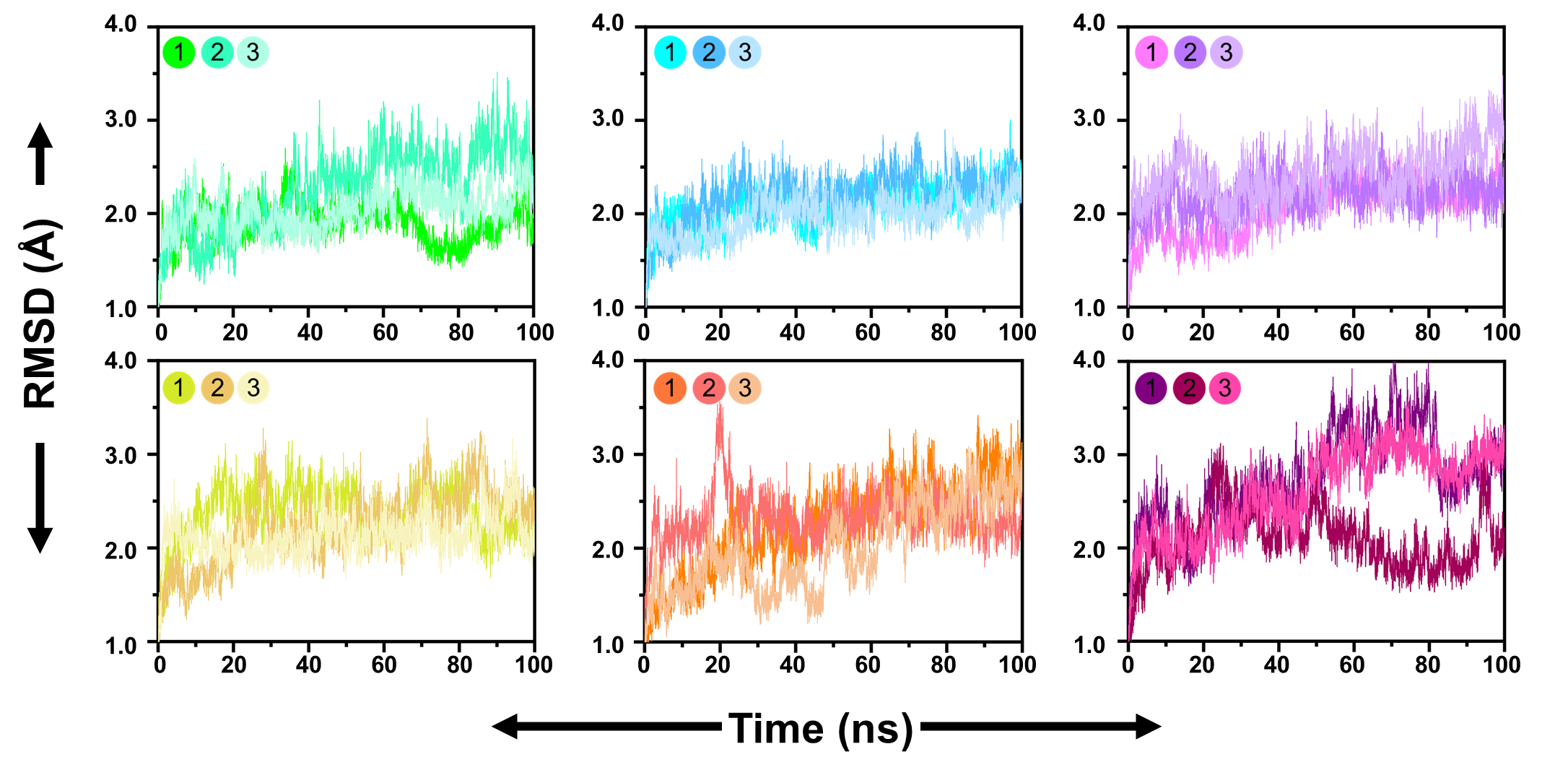


**Figure S2.** The triplicates of compound-RBD complex RMSD analysis. The average RMSD of ACE-RBD (green), ISO-RBD (pink), HYP-RBD (cyan), CAG-RBD (yellow), ORO-RBD (orange) and CGA-RBD (purple) were 2.1±0.2, 2.1±0.1, 2.2±0.2, 2.2±0.1, 2.2±0.2 and 2.5±0.3 Å, respectively.


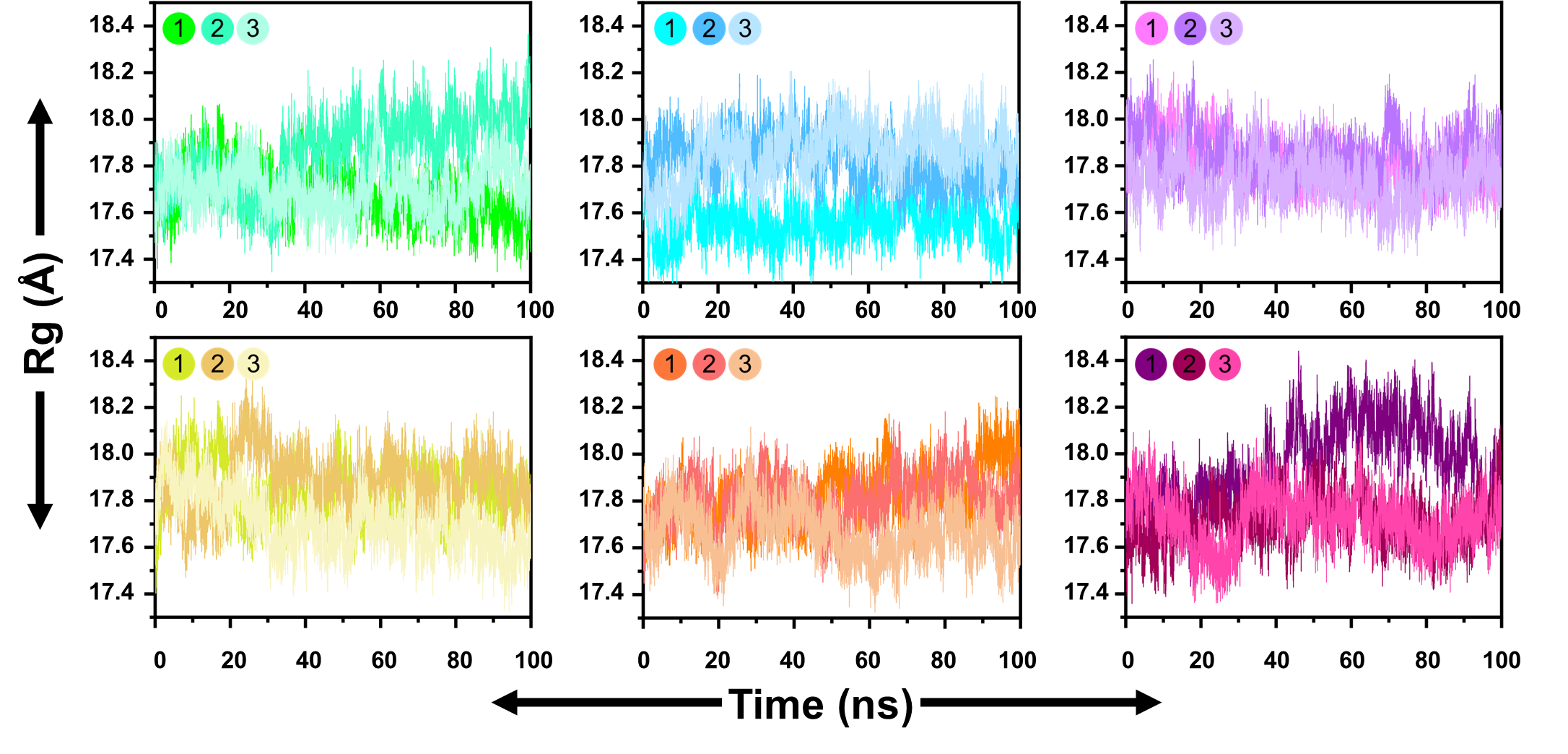


**Figure S3.** The triplicates of Radius of gyration (Rg) during MD simulations. Rg plot of ACE-RBD (green), ISO-RBD (pink), HYP-RBD (cyan), CAG-RBD (yellow), ORO-RBD (orange) and CGA-RBD (purple) docked complexes with the average value (Å) of 17.7±0.1, 17.7±0.2, 17.8±0.1, 17.8±0.1, 17.8±0.1 and 17.8±0.1 Å, respectively.


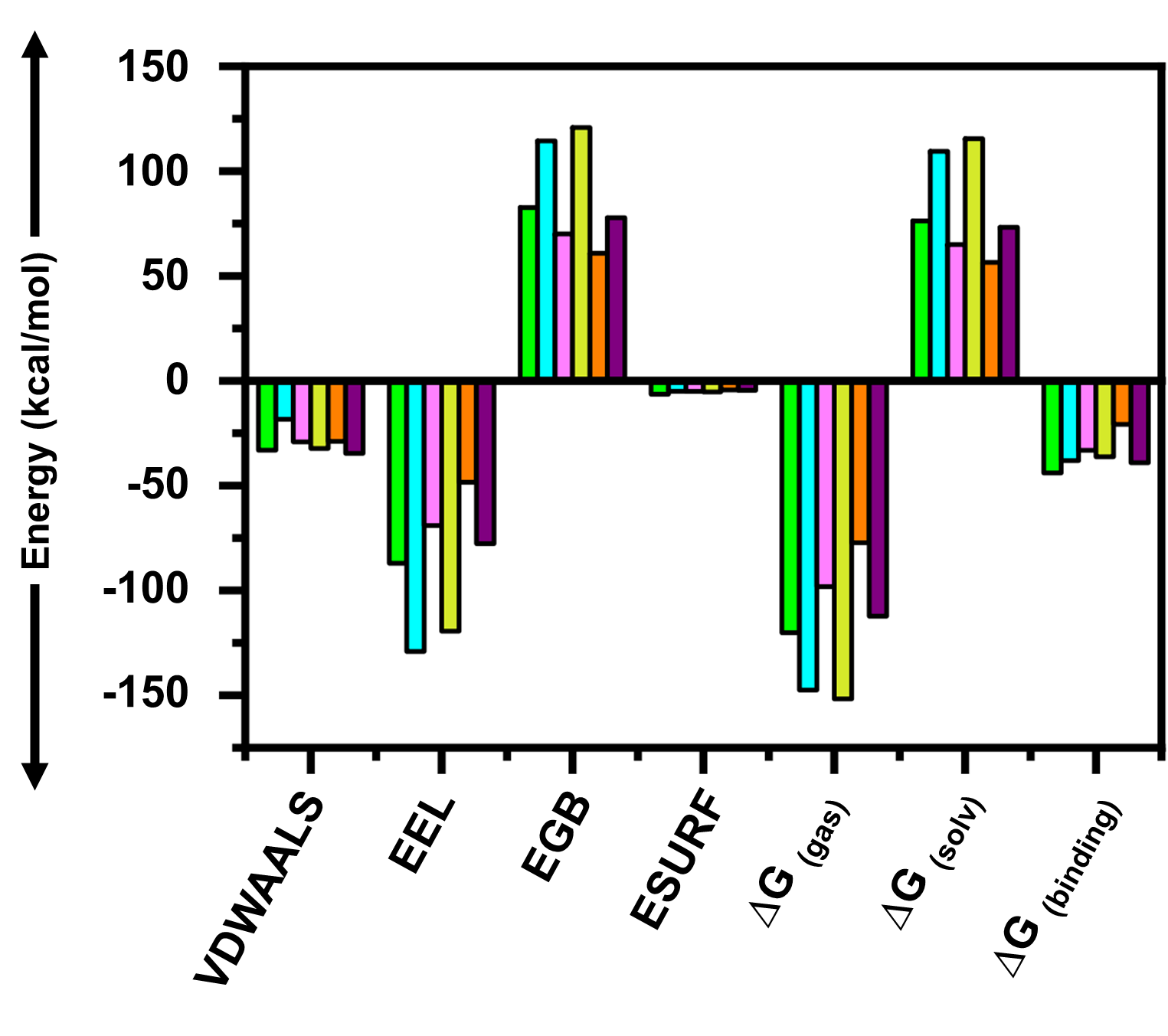


**Figure S4** Decomposition of binding free energy. ACE-RBD (green), HYP-RBD (cyan), ISO-RBD (pink), CAG-RBD (yellow), ORO-RBD (orange) and CGA-RBD (purple). VDWAALS = van der Waals contribution from MM. EEL = electrostatic energy as calculated by the MM force field. EGB = the electrostatic contribution to the solvation free energy calculated by GB. ESURF = The surface area energy.

**Table S1.** The force field, charge parameter and SMILES format for natural compounds in this study.

| **Compound** | **Force field** | **Charge parameter** | **SMILES format** |
| --- | --- | --- | --- |
| ACE | gaff2 | 0 | CC1C(C(C(C(O1)OC2C(C(OC(C2OC(=O)C=CC3=CC(=C(C=C3)O)O)CO)OCCC4=CC(=C(C=C4)O)O)O)O)O)O |
| HYP | gaff2 | 0 | C1=CC(=C(C=C1C2=C(C(=O)C3=C(C=C(C=C3O2)O)O)OC4C(C(C(C(O4)CO)O)O)O)O)O |
| ISO | gaff2 | 0 | C1=CC(=C(C=C1C2=C(C(=O)C3=C(C=C(C=C3O2)O)O)OC4C(C(C(C(O4)CO)O)O)O)O)O |
| CAG | gaff2 | 0 | C1C(C(C(C(O1)C2=C(C(=C3C(=C2O)C(=O)C=C(O3)C4=CC=CC=C4)C5C(C(C(C(O5)CO)O)O)O)O)O)O)O |
| ORO | gaff2 | 0 | COC1=C(C=C2C(=C1O)C(=O)C=C(O2)C3=CC=CC=C3)OC4C(C(C(C(O4)C(=O)O)O)O)O |
| CGA | gaff2 | 0 | C1C(C(C(C(O1)C2=C3C(=C(C(=C2O)C4C(C(C(C(O4)CO)O)O)O)O)C(=O)C=C(O3)C5=CC=CC=C5)O)O)O |
